# Editorial: Nano-Bio Interactions: Ecotoxicology and Cytotoxicity of Nanomaterials

**DOI:** 10.3389/fbioe.2020.00918

**Published:** 2020-09-16

**Authors:** Jianbo Jia, Tania Limongi, Yin Liu, Gaoxing Su, Hongyu Zhou

**Affiliations:** ^1^Key Laboratory of Pearl River Delta Water Quality Safety and Protection, Ministry of Education, Institute of Environmental Research at Greater Bay, Guangzhou University, Guangzhou, China; ^2^Department of Applied Science and Technology, Polytechnic University of Turin, Turin, Italy; ^3^Department of Pharmaceutical Sciences, Irma Lerma Rangel College of Pharmacy, Texas A&M University, College Station, TX, United States; ^4^Research Center for Eco-Environmental Sciences (CAS), Beijing, China; ^5^School of Pharmacy, Nantong University, Nantong, China

**Keywords:** engineered nanomaterials (ENMs), cytotoxicity, nano-bio interaction, nanotoxicology, nanomedicine

The past few decades have witnessed a boom in nanotechnology in various areas, raising global concerns about the environmental health and safety of engineered nanomaterials (ENMs). It has always been a challenging task to grasp the full picture of nanotoxicology and nanoecotoxicology, as multiple parameters should be taken into consideration for assessments simultaneously, including the physicochemical properties of ENMs, the migration and transformation of ENMs in the environment and organisms, and the physiological and pathological conditions of the testing organisms. This topic intends to explore nano-bio interactions from the aspects of ecotoxicology and cytotoxicity of ENMs, so as to efficiently modulate their bioactivities and reduce the potential risks.

The Research Topic “Nano-Bio Interactions: Ecotoxicology and Cytotoxicity of Nanomaterials” includes three reviews, one systematic review, and one original research, with the purpose of maximizing the understanding of how ENMs' cellular internalization process affects cell damages and deaths, which are related to their ecotoxicology and cytotoxicity. The fascinating properties of two-dimensional transition metal dichalcogenides (2D TMDCs) make them highly attractive for applications ranging from electronics to nanomedicines. For a better understanding of 2D TMDCs, two kinds of synthesis and surface functionalization methods (top-down and bottom-up) have been reviewed by Zhou et al.. The recent research progress of 2D TMDCs with particular focus on their biomedical applications and potential health risks are discussed. Authors also emphasize how 2D TMDCs interacting with biological systems can sometimes interfere with normal cellular functions, the metabolic processes, and cell death. More in detail, apart from 2D TMDCs' chemical composition, size, and surface modification, they underline how their thickness effectively contributes to the establishment of cytotoxic effects even without entering cells. In another review of this special issue, Sun et al. summarized the effects of surface chemistry on various cytotoxicity-related bioactivities induced by ENMs. Negatively charged nanoparticles have a high protein binding capacity, whereas positively charged and hydrophobic nanoparticles can be more easily internalized by cells, triggering oxidative stress induction and the related autophagy, necrosis, and/or apoptosis phenomena. However, PEG decoration can counteract these cytotoxicity-related bioactivities. In their review, Sun et al. recommend surface decoration by combinatorial chemistry methods as an effective strategy to reveal the impact of surface chemistry on nanotoxicology. Besides, specific molecular pathways regulated by surface chemistry of ENMs are also discussed.

As one of the most important parameters in understanding possible health issues of ENMs, it is necessary to clarify the role of the “protein corona” in the interactions between NPs and physiological systems, such as biological media and blood. Yu et al. explored the effects of surface chemistry on the formation and dynamic behavior of the protein corona using a gold nanoparticle array with a wide range of surface hydrophobicities. Generally, nanoparticles coated by high hydrophobic surfaces adsorb smaller and negatively charged proteins, and exhibit a lower hard corona protein exchange rate than their hydrophilic counterparts. The types of adsorbed proteins are also affected by the hydrophobicity of ENMs. These findings enhance our understanding of protein corona formation and protein exchange dynamics, and will help researchers in the modulation of nanotoxic effects by controlling protein corona formation through surface NPs modifications.

As discussed by Gedda et al. ENMs may induce cytotoxicity through epigenetic changes at DNA, RNA, and protein levels. ENMs are considered as epimutagens, as they can promote neoplastic changes by disrupting the function of epigenetically preserved genes via global epigenetic processes. They may induce epigenetic toxicity by altering DNA methylation profiles, changing histone modifications, and regulating non-protein-coding RNAs at sub-cytotoxic and sub-genotoxic ENM concentrations. Although numerous “toxico-omics” studies have been conducted on ENMs, a specific “risk assessment paradigm” dealing with the epigenetic modulations in humans owing to the exposure of ENMs has not yet been defined. Thus, Gedda et al. propose that constructing a universally recognized and centralized database concerning reproducible ENMs' characterization and *in vivo* standardized evaluation on ENMs' epigenetic toxicity can be considered a benefit in understanding the nature and extent of NPs nanotoxicity.

As for biological systems used for nanotoxicity assessments, two-dimensional (2D) cell models can hardly reflect the complex tissue microenvironments and interactions between ENMs and different cells types, while animal models are not suitable for high-throughput toxicity screening. Three-dimensional (3D) cell models have been used as an improved cell-based assay with more biological relevance and as an alternative method to animal experimentation. In a systematic review, Sanches et al. assessed whether 3D skin models can be considered as an appropriate evaluation method of the TiO_2_ nanoparticles toxicity. Through the evaluation of the reliability of data extracted from seven selected articles, they conclude that 3D skin models are suitable for testing the hazard effect of TiO_2_ nanoparticles, while emphasizing the need of standardized protocols for the related risk assessment procedures.

Taken together, although this topic included some very recent advances in the fields of nanotoxicity and nanoecotoxicology, challenges are still overwhelming, such as ever-increasing production, usage and disposal of ENMs and their based products, developing standardized high-throughput nanotoxicity testing protocols, establishing centralized database on nanotoxicity, and developing intelligent prediction models of all nano-bio interactions. To conclude, addressing these challenges is fundamental for a better understanding of nanotoxicity and for the design of the next generation of ENM with a better reliability and safety profile.

## Author Contributions

All authors listed have made a substantial, direct and intellectual contribution to the work, and approved it for publication.

## Conflict of Interest

The authors declare that the research was conducted in the absence of any commercial or financial relationships that could be construed as a potential conflict of interest.

